# Vapor-Phase-Deposited Ag/Ir and Ag/Au Film Heterostructures for Implant Materials: Cytotoxic, Antibacterial and Histological Studies

**DOI:** 10.3390/ijms25021100

**Published:** 2024-01-16

**Authors:** David S. Sergeevichev, Svetlana I. Dorovskikh, Evgeniia S. Vikulova, Elena V. Chepeleva, Maria B. Vasiliyeva, Tatiana P. Koretskaya, Anastasiya D. Fedorenko, Dmitriy A. Nasimov, Tatiana Y. Guselnikova, Pavel S. Popovetsky, Natalya B. Morozova, Tamara V. Basova

**Affiliations:** 1 Nikolaev Institute of Inorganic Chemistry SB RAS, 3 Lavrentiev Ave., Novosibirsk 630090, Russia; d_sergeevichev@meshalkin.ru (D.S.S.); reter16@yandex.ru (S.I.D.); lazorevka@mail.ru (E.S.V.); pantel@niic.nsc.ru (T.P.K.); fedorenko@niic.nsc.ru (A.D.F.); tguselnikova@niic.nsc.ru (T.Y.G.); popovetskiy@niic.nsc.ru (P.S.P.); mor@niic.nsc.ru (N.B.M.); 2 NMRC Named after Academician E.N. Meshalkin of the Ministry of Health of the Russian Federation, 15, Rechkunovskaya St., Novosibirsk 630055, Russia; amareza@mail.ru (E.V.C.); vasilievam@yandex.ru (M.B.V.); 3 V. Zelman’s Institute of Medicine and Psychology, Novosibirsk State University, 2, Pirogov St., Novosibirsk 630090, Russia; 4 Rzhanov Institute of Semiconductor Physics SB RAS, 13 Lavrentiev Ave., Novosibirsk 630090, Russia; nasimov@isp.nsc.ru

**Keywords:** implant, titanium-alloy, CFR-PEEK, iridium, gold, silver, vapor deposition, thin films, antibacterial activity, histological study

## Abstract

Using gas-phase deposition (Physical Vapor Deposition (PVD) and Metal Organic Chemical Vapor Deposition (MOCVD)) methods, modern implant samples (Ti alloy and CFR-PEEK polymer, 30% carbon fiber) were functionalized with film heterostructures consisting of an iridium or gold sublayer, on the surface of which an antibacterial component (silver) was deposited: Ag/Ir(Au)/Ti(CFR-PEEK). The biocidal effect of the heterostructures was investigated, the effect of the surface relief of the carrier and the metal sublayer on antibacterial activity was established, and the dynamics of silver dissolution was evaluated. It has been shown that the activity of Ag/Ir heterostructures was due to high Ag^+^ release rates, which led to rapid (2–4 h) inhibition of *P. aeruginosa* growth. In the case of Ag/Au type heterostructures, the inhibition of the growth of *P. aeruginosa* and *S. aureus* occurred more slowly (from 6 h), and the antibacterial activity appeared to be due to the contribution of two agents (Ag^+^ and Au^+^ ions). It was found, according to the in vitro cytotoxicity study, that heterostructures did not exhibit toxic effects (cell viability > 95–98%). An in vivo biocompatibility assessment based on the results of a morphohistological study showed that after implantation for a period of 30 days, the samples were characterized by the presence of a thin fibrous capsule without volume thickening and signs of inflammation.

## 1. Introduction

Silver nanoparticles (AgNPs) have a number of unique physicochemical properties and are widely used in the development and production of new-generation materials. Due to the high biocidal activity against about 650 strains of pathogenic microorganisms [1], AgNPs and Ag-containing coatings are used in biological and biomedical technologies [2]. In this regard, an important direction is to improve modern implant biomaterials to solve the problem of peri-implant inflammation and infections [3], which can lead to repeated operations [4]. This is especially dangerous for patients with weakened immune systems: the frequency of infections of endoprostheses in cancer patients reaches 66% [5]. Infections are usually associated with the growth of bacterial biofilms on the surface of implants that protect pathogenic microorganisms from the host’s defense mechanisms, which can cause serious complications in the short and long term after the introduction of the biomaterial into the body.

The long-term antimicrobial activity of materials can be ensured by the presence of components on the surface, which inhibit the growth of bacterial colonies and at the same time do not have a toxic effect on surrounding tissues. Studies of the antimicrobial properties of AgNPs and coatings deposited on the surface of titanium and its alloys (one of the traditionally used implants) have confirmed the high antibacterial activity of the modified materials compared with the unmodified ones [6,7]. The activity of Ag-containing film structures depends on the type of strains, the content and shape of the component, including particle sizes, layer thickness, and the chemical composition of the material surface [8,9]. In fact, the mechanism of action may combine several aspects, such as destruction of the bacterial cell membrane, blocking of active respiratory chains and/or enzymes of nutrient transport through the cell membrane, and generation of reactive oxygen species (ROS) [10,11,12,13]. Although in the general case, both the nanoscale and chemical effects are considered here, the temporal dynamics of the action is largely determined by the nature of the release of Ag^+^ ions, which occurs due to the oxidation of metal particles/grains on the surface during their interaction with contacting liquids. Thus, it is possible to provide a prolonged antibacterial effect by controlling the dynamics of oxidative dissolution of silver, which is important for medical practice [14,15].

It is important that the concentration of Ag ions released from the material provides the necessary antimicrobial efficacy (from 0.1 ppb) [16] and is constantly below the level of toxicity to human cells [17]. Moreover, the effect of possible Ag accumulation should be taken into account in the biomedical application of these materials in order not to exceed the safe dose for human [18,19]. It was shown in previous works [20,21] that AgNPs are cytotoxic for some cell types, for example, mononuclear cells of human peripheral blood. The mechanism and criteria of toxicity are still the subject of research, but it is already clear that the concentration and size of AgNPs are important factors. Taking into account the equilibrium between the various forms of silver in biological solution (nano- and ultra-particles, chemically bound ions), the results concerning the biocompatibility of silver nanolayers and AgNPs require further clarification. In this regard, the study of the body/tissue reaction in vivo is an important direction.

On the other hand, to significantly improve the biocompatibility of the material, i.e., to prevent inflammatory reactions, the individual action of silver in any form is usually insufficient. The use of composite Ag-containing materials has been shown to be the most effective approach [7,14,15,22]. For example, it has recently been shown that due to the galvanopair effect, the combination of silver with less active noble metals (platinum, palladium, iridium, gold) makes it possible to achieve a significant increase in the antibacterial effect [23], which persists even under conditions of reduced diffusion. It was found in our previous studies [24,25,26] that such film heterostructures with an active surface also improve in vivo biocompatibility of modern implant materials (metal, polymer, carbon). It has also been shown that it is possible to control both the dynamics of antibacterial activity and the character of the material survival rate by varying the rate of dissolution of Ag from the surface of platinum metal (Pt or Ir) [24,26].

Although the literature abounds with many publications on the activity of metal nanoparticles, including Ag, Au, and their composites [5,7,13,14,15,22], studies of film heterostructures based on noble metals are sporadic. The effect of platinum group metals and gold on the release of silver deposited on the surface of Ir, Pt, Pd, and Au in the form of nanodots was studied by Köller and co-authors [23,27]. The samples were prepared via magnetron sputtering and a lithographic process. It was shown that the most effective antibacterial pair was Ag/Ir that demonstrated complete inhibition of bacterial growth within 24 h, which correlated with the fastest dissolution of silver nanodotes and the maximum release of Ag. In our previous work [5], we studied the antibacterial and cytotoxic properties of heterostructures in which Ag and Au nanoparticles and clusters were deposited onto the surfaces of modern implant materials covered with Ir, Pt, and IrPt alloy. PVD and CVD methods were used to prepare the heterostructures.

Despite the success achieved in this new direction, a number of aspects remain unexplored. First of all, it is necessary to study the effect of the concentration and form of the active component (silver) on the surface in more detail and to determine the most effective noble metal in such a heterostructure. It was shown in our previous studies [24,25,26], using of Pt- and Ir-based materials as examples, that the application of bimetallic alloys was unreasonable because it caused a delayed toxic effect in vivo [24]. As mentioned above, Köller et al. [27] demonstrated the possibility of using gold as a sublayer for the heterostructures. In general, gold surfaces can stimulate the growth of fibroblast cells, improving the survival process [28].

Among the various methods used for the synthesis of Ag-containing film structures, including solution, photochemical, physical vapor deposition (PVD), and chemical vapor deposition (MOCVD), the latter two methods have significant advantages that determine their choice [5]. Gas-phase methods provide precision control of the processes of nucleation and growth of metal layers with certain composition, structure, morphology, physico-chemical and biological properties [29,30,31]. It was noted [32] that in gas-phase processes, silver tends to nucleate and grow in the form of islands according to the Volmer–Weber growth mode, which is favorable for the formation of particles/clusters/discontinuous layers. This mechanism is probably realized due to a stronger interaction between silver atoms compared to the silver-substrate interaction, which leads to the formation of islands of metal nanoparticles on the coated surface. In addition, in this case, the absence of foreign chemical compounds, which can be included in the composite obtained by solution methods and affect the overall biological response of the system, is practically guaranteed. Thus, gas-phase methods make it possible to achieve the best direct contact of silver with the noble metal, which ensures the effective action of galvanopairs.

Thus, the present work is aimed at comparing iridium and gold as sublayers on the surface of which an antibacterial component (silver) was deposited for the formation of heterostructures to improve implant materials. To study the effect of deposition conditions on the characteristics of the layers, the layers were obtained by two gas-phase methods, namely PVD and MOCVD. The biocidal effect and cytotoxicity of the heterostructures deposited onto biomaterials, as well as their histological parameters after implantation in experimental animals were investigated.

## 2. Results

**Figure 1:**
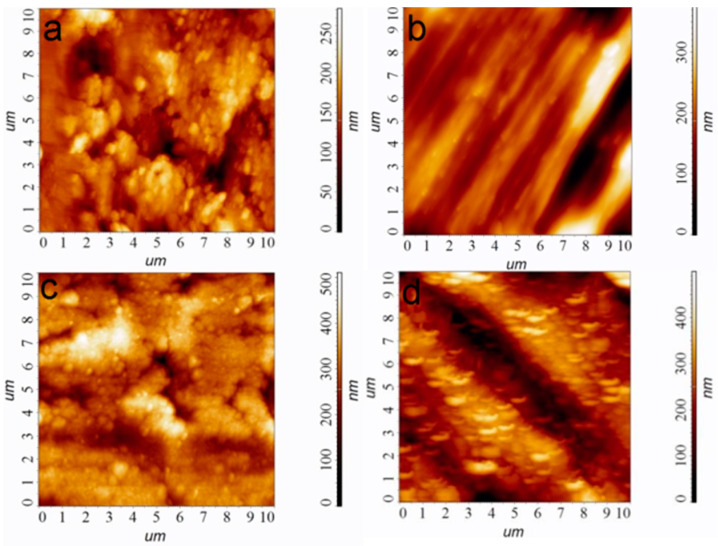
AFM images of the sample surfaces, bare Ti (**a**), bare Ti* (**b**), Ir/Ti (**c**), and Ir/Ti* (**d**). Scale is 10 × 10 µm.

A series of heterostructures were obtained for the current study. The heterostructures consisted of a carrier biomaterial, on which, first, the sublayer of metal (Ir or Au) and then the antibacterial component Ag (on the top of the sublayer) were deposited. The following carriers were used as implant biomaterials:

- “Ti” carriers: pristine Ti6Al4V discs; according to atomic force microscopy (AFM) data, the average roughness (R_a_) is 30 nm (Figure 1a);
- “Ti*” carriers: surface-polished Ti6Al4V discs; according to AFM data, the ordered grooved altitude differences are up to 300 nm whereas R_a_ is 60 nm (Figure 1b);
- “CFR-PEEK” carriers: polyetheretherketone discs reinforced with carbon fiber (30%); according to AFM data, R_a_ is 55 nm.

### 2.1. Deposition and Characterization of Ir and Au Sublayers

Ir and Au were selected as “bottom” layers of heterostructures to modify the implant materials because they are the most inert of the noble metals capable of creating effective galvanopairs with Ag. Effective MOCVD (Ir) and PVD (Au) processes developed in our previous works [26,33] were used to obtain these layers.

Iridium layers were prepared via MOCVD, using well-developed procedures according to our previous work [26]. Specific conditions for obtaining Ir/Ti, Ir/Ti*, Ir/CFR-PEEK samples ensuring Ir layer thickness 1.0 ± 0.1 μm are given in Par. 4.2. According to atomic force microscopy (AFM) data, and the noble coatings repeat the surface relief; however, after iridium covering, the average roughness Ra values increases by 30–50% (Figure 1c,d). This is due to the developed columnar-dendritic microstructure of the Ir layers formed under the selected deposition conditions.

**Figure 2:**
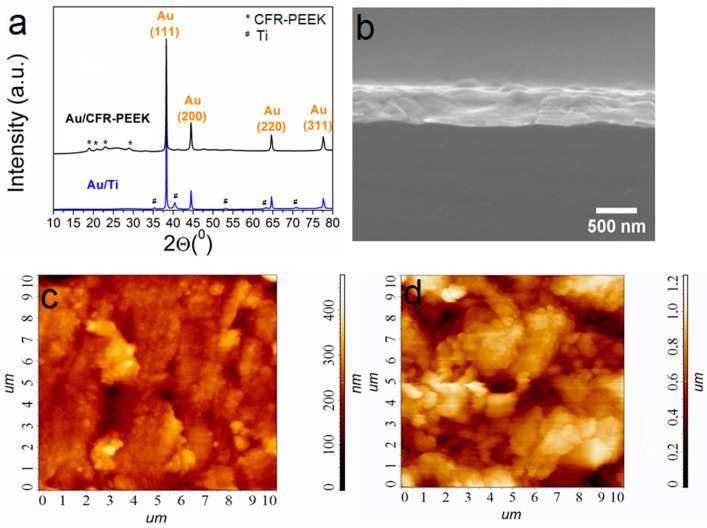
XRD patterns of Au/Ti and Au/CFR-PEEK, where the unlabeled peaks belong to the carrier materials (**a**); cross-section SEM image of Au/Si (**b**); AFM images of Au/Ti (**c**) and Au/CFR-PEEK (**d**). Scale is 10 × 10 µm.

Gold layers were prepared using a thermal PVD process specially developed based on our recent research [33]. Specific conditions for obtaining Au/Ti and Au/CFR-PEEK samples are given in Section 4.2. Typical X-ray diffraction (XRD) patterns of the gold-coated biomaterials of the samples are shown in Figure 2a. Both Au/Ti and Au/CFR-PEEK are formed by a single crystalline fcc-Au phase (2θ = 38.1°(111), 44.4°(200), 64.5°(220), 77.7°(311) [34]). Low intense reflexes related to Ti (*P*63/mmc, 2θ = 35.1°(100), 40.5°(101), 53°(102), 63.1°(110), 71°(103)) [35] or CFR-PEEK (2θ = 18.8(110), 20.8(111), 22.9(200), 28.8°(211)) [36] are also visualized on XRD patterns of biomaterials with gold layers. The Au coatings are textured in the direction (111) which is characteristic for thermal PVD of gold [37]. Regardless of the type of carrier, the gold crystallite size estimated from XRD data is 40(4) nm. Scanning electron microscopy (SEM) analysis of cross-sections of samples on model Si substrates showed that 100 mg of an initial gold powder provides an Au layer thickness of 0.8 ± 0.1 μm (Figure 2b). Such grains form porous layers, and on the surface they are grouped into elongated agglomerates with sizes of 200–500 nm (Figure 2c,d). According to AFM, the average roughness R_a_ is 40 nm for Au/Ti and 180 nm for Au/CFR-PEEK (Figure 2c,d).

### 2.2. Deposition and Characterization of Ag/Ir and Ag/Au Heterostructures

The final step in the preparation of the heterostructures was the deposition of an antibacterial component (Ag) onto the surface of the iridium- or gold-coated biomaterials. To determine the effect of the method of deposition of the Ag antibacterial component on the characteristics of film heterostructures, CVD and PVD methods were tested.

To vary the silver content in the resulting samples sequential deposition procedures were used (Section 4.2). According to previous results [26], Ag surface concentrations of no more than 50 μg/cm^2^ at which coatings do not exhibit pronounced cytotoxicity were selected to prepare heterostructure in this work.

#### 2.2.1. Ag/Ir and Ag/Au Heterostructures Obtained via the PVD Method

*General deposition features*

To prepare the heterostructure via PVD, one, two, and three consecutive PVD procedures were carried out under the conditions described in Section 4.2. According to the quantitative ICP-OES data, the material of the sublayer (Ir or Au) and the carrier material (Ti or CFR-PEEK) do not affect the silver content in the obtained heterostructures. The proposed approach provides control of the Ag content on the surface: the dependence of Ag content on the number of PVD procedures is almost linear (Pearson coefficient is 0.999, Figure S1, Supporting Information (SI)). The surface concentration of silver varies in the range of 6–20 µg/cm^2^. Silver is present on the surface of noble metals in two forms (Figure S2). After one deposition procedure (Figure S2a), Ag films (~5 nm thick) with separate AgNPs (5–20 nm) are formed. Then the film thickness increases, and the particles form clusters up to 40 in size (two deposition procedures, Figure S2b) or 100 nm (three deposition procedures, Figure S3b).

**Figure 3:**
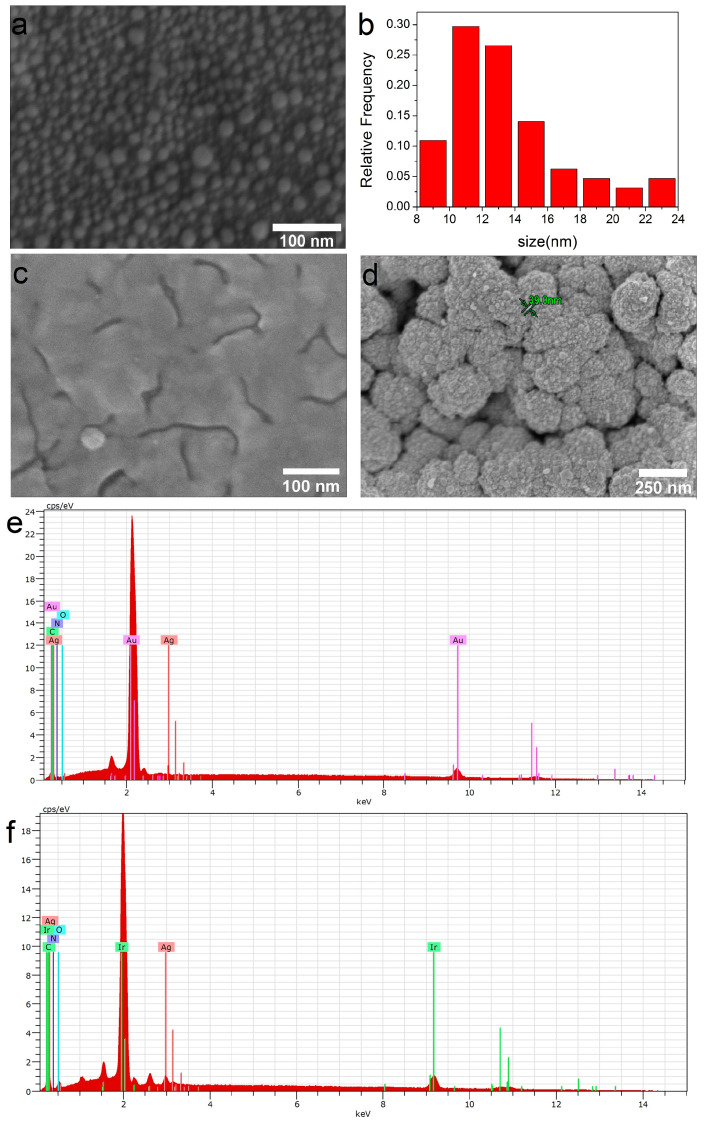
SEM micrographs of the samples obtained via PVD: Ag/CFR-PEEK (**a**) and corresponding AgNPs size distribution (**b**); Ag/Au/CFR-PEEK (**c**); Ag/Ir/CFR-PEEK (**d**); EDX spectra of Ag/Au/CFR-PEEK (**e**); Ag/Ir/CFR-PEEK (**f**).

It should be noted that under similar conditions, silver films do not form on the surface of bare biomaterials. A comparison of samples obtained after two consecutive PVD procedures is presented in Figure 3. Only isolated AgNPs with sizes of 5–25 nm are observed on the surface of both CFR-PEEK and Ti (Figure 3a,b).

*Characterization of the samples for biological studies*

Due to the fact that the composition of heterostructures does not depend on the carrier, CFR-PEEK samples were selected for biological studies from this series. Samples obtained by two consecutive PVD procedures, i.e., with a silver content of ~13 µg/cm^2^, were examined. In such Ag/Au and Ag/Ir heterostructures, the thickness of the silver film is ~7 nm. Individual AgNPs (5–20 nm) or agglomerates of AgNPs (40–60 nm) can also be detected on the crystallites of the sublayer (in the case of Ir) or on their boundaries (in the case of Au) (Figure 3c,d). According to EDX data (Figure 3e,f), the silver content in Ag/Au and Ag/Ir heterostructures lies in the range of 1.4–1.9 at.%.

**Figure 4:**
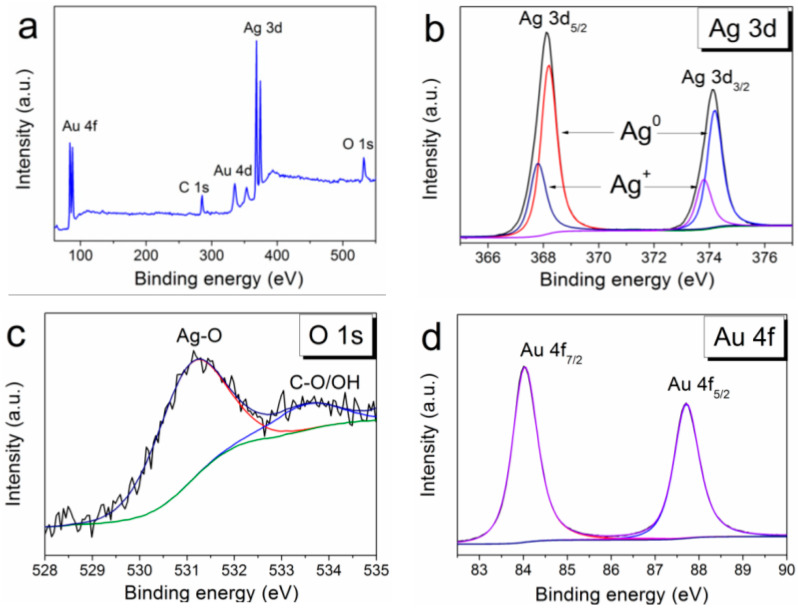
XPS spectra of Ag/Au/CFR-PEEK (**a**), fitting of Ag3d spectra (**b**), fitting of O1s spectra (**c**), and fitting of Au4f spectra (**d**).

The detailed data on the chemical composition of PVD-formed Ag/Ir heterostructures obtained by the method of X-ray photoelectron spectroscopy (XPS) were presented in our previous work [25]. Both components of the heterostructure were detected only in the metallic state. The XPS surface survey spectrum of the Ag/Au/CFR-PEEK sample is shown in Figure 4a. The analysis of the spectra shows that silver is present in both the metallic and oxidized states with the corresponding binding energies Ag^0^ 3d_5/2_ = 368.3 eV and Ag^+^ 3d_5/2_ = 367.8 eV эB (Figure 4b). In quantitative terms, the metallic phase prevails (Ag^0^/Ag^+^ = 4). Oxygen content decreases during Ar^+^ (1 keV) etching from 18 to 3 at. % (after 120 s of etching). However, the peak corresponding to Ag-O is constantly present in the O1s spectra (Figure 4c). Gold is present only in the metallic state (4f_7/2_ = 84.0 eV) (Figure 4d).

#### 2.2.2. Ag/Ir and Ag/Au Heterostructures Obtained via MOCVD

*General deposition features*

Similar to the PVD process, one, two, and three consecutive MOCVD procedures were also performed under the conditions described in Section 4.2. The dependence of the deposited silver amount on the number of cycles also turned out to be close to linear (Pearson coefficient 0.996, Figure S1). The resulting silver contents varied in the range of 15–45 µg/cm^2^ with the larger error bars (Figure S1).

The features of the sequential MOCVD deposition of silver on noble metal surfaces are similar to those described above (Section 2.2.1). After the first deposition procedure (Figure S3a), Ag films (~5–7 nm thick) with separate AgNP clusters are formed on the surface of noble metals. Further, the film thickness practically does not change, an enlargement of Ag clusters (almost twofold, up to 80 nm) and their agglomeration into film islands are observed (Figure 3b,c). Similar to the PVD process, a silver film does not form on the surface of pristine biomaterials during MOCVD, and the sizes of nanoparticles/clusters are expected to increase during sequential deposition.

*Characterization of the samples for biological studies*

**Figure 5:**
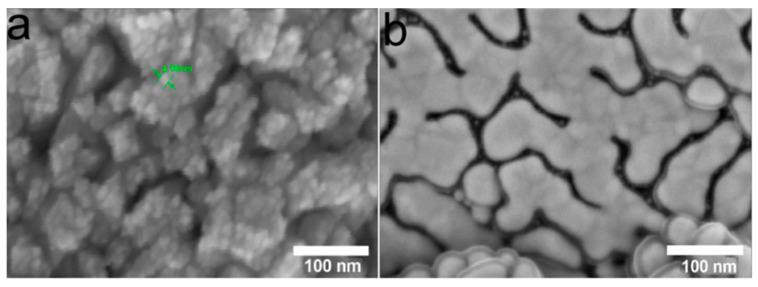
SEM micrographs of the Ag/Ir/Ti (**a**) and Ag/Au/Ti (**b**) samples obtained via MOCVD.

Since the compositions of heterostructures do not depend on the carrier, samples on Ti were selected for biological studies from this series. Samples obtained after one deposition cycle, i.e., with a silver content of 15–18 µg/cm^2^, were studied. In such Ag/Au and Ag/Ir heterostructures, the thickness of the silver film is ~5–7 nm (Figure 5, dark gray areas), and the sizes of separate AgNP clusters/agglomerates vary from 20 to 40 nm (Figure 5).

**Figure 6:**
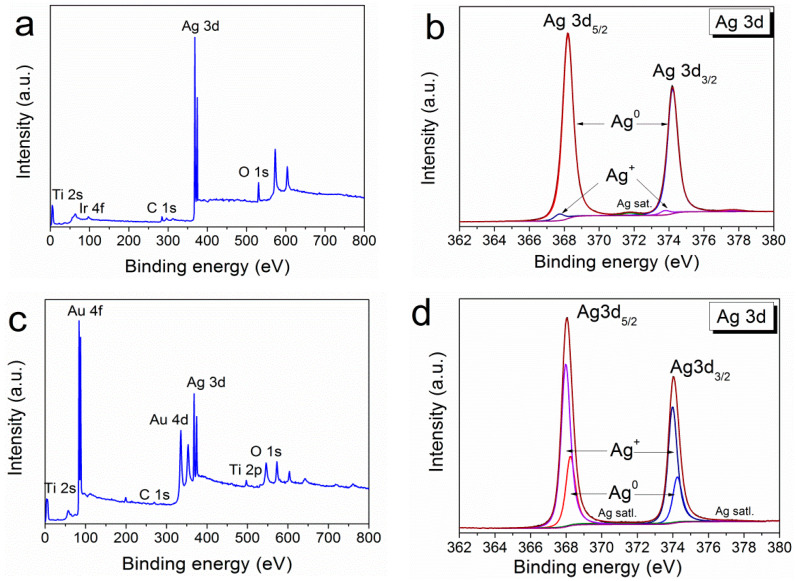
XPS survey spectra of Ag/Ir/Ti (**a**) with the fitting of Ag3d spectra (**b**) and XPS survey spectra of Ag/Au/Ti (**c**) with the fitting of Ag3d spectra (**d**).

Since the processes of MOCVD of silver on noble metals have been little studied [25], Figure 6 shows XPS surface survey spectra for both types of heterostructures (Ag/Ir and Ag/Au). Oxidized silver was found to be present in both cases (Figure 6b,d). In the Ag/Au heterostructure, the oxidized phase becomes dominant (Ag^0^:Ag^+^ = 1:2.5) while its amount is small (Ag^0^:Ag^+^ = 10:1) for Ag/Ir. The ratio of these components changes slightly during Ar^+^ etching.

### 2.3. Biological Studies

#### 2.3.1. Antibacterial Activity

To investigate the antibacterial activity, Ag/Au/Ti, Ag/Ir/Ti, Ag/Ir/Ti*, and Ag/Ir/CFR-PEEK samples with surface silver concentrations of 13.2–18.0 μg/cm^2^ were prepared. Each series consists of three experimental samples obtained under the same conditions (see details in Section 4.2). The biocidal efficacy of the heterostructures was investigated using *Staphilococcus aureus* (G+ bacterium), which is the main causative agent of peri-implant infections [38], and *Pseudomonas aeruginosa* (G− bacterium), which is characterized by high resistance to antibiotics and the percentage of treated cancer patients [39]. The study was conducted using the direct contact method by applying a certain number of CFU to the sample surface and counting the surviving bacterial colonies by light microscopy. To evaluate the dynamics of the effect, the bacterial cultures were incubated on the surface of the samples for 2, 4, 6, 24, and 48 h. Numerical data on the number of surviving bacterial colonies at each time interval are summarized in Table S1.

**Figure 7:**
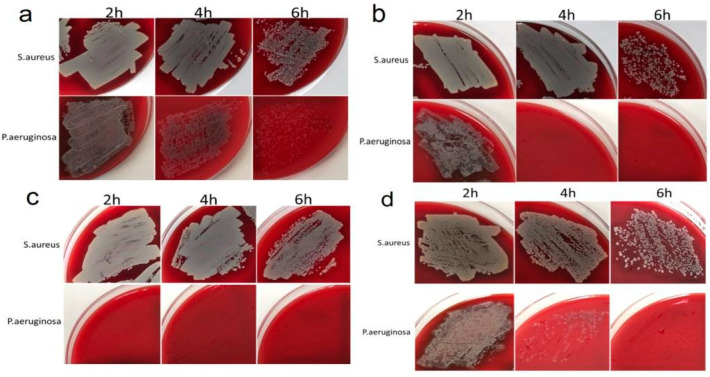
Antibacterial activity profile at 2, 4, and 6 h of Ag/Au/Ti (**a**), Ag/Ir/Ti (**b**), Ag/Ir/Ti* (**c**), and Ag/Ir/CFR-PEEK (**d**) samples against *S. aureus* or *P. aeruginosa*.

Importantly, all samples completely inhibited bacterial growth after 24 h of incubation (Table S1). In the absence of heterostructures (control measurements, see details in Section 4.4.1), the growth of both bacterial colonies was observed in Petri dishes after 24 h of incubation (Figure S4). At the same time, marked differences were observed at shorter exposure times (Figure 7). In particular, the growth inhibition of *P. aeruginosa* at 2–4–6 h exposure time was generally strong, ranging from 10–55–85% (Ag/Au/Ti) and 5–85–100% (Ag/Ir/CFR-PEEK) to 20–100–100% (Ag/Ir/Ti sample) and 100–100–100% (Ag/Ir/Ti* sample). For *S. aureus*, a similar pattern was less active, ranging from 0–2–70% (Ag/Ir/Ti sample) and 0–5–50% (Ag/Ir/Ti* sample) to 2–10–60% (Ag/Ir/CRF-PEEK sample) and 3–17–75% (Ag/Au/Ti sample).

The effect of surface roughness on the antibacterial performance of the film heterostructure is demonstrated by comparing the data for the Ag/Ir/Ti and Ag/Ir/Ti* series of experiments. In the case of *P. aeruginosa*, the quantitative biocidal effect over 2 h is different: 20% (heterostructure on standard Ti surface) and 100% (Ag/Ir/Ti* analog with doubled roughness). However, both series continue to show complete inhibition. In the case of *S. aureus*, a noticeable biocidal effect of the samples is observed only after 6 h, with two different effects. Complete inhibition is also observed. These differences appear to be due to an increase in the dissolution rate of Ag from a more developed surface.

The effect of the continuous layer material is also manifested in the case of gram-negative *P. aeruginosa*, and the biocidal effect is significantly more pronounced for Ir-containing heterostructures. In particular, Ag/Ir/Ti samples show 100% inhibition within 4 h, while Ag/Au/Ti samples do not show more than 50% inhibition. On the contrary, no such “selective” effect is observed for *S. aureus*, i.e., the dynamics of Ag/Au and Ag/Ir heterostructures are similar.

In the previous study, we showed correlations between the dynamics of antibacterial activity of Ag/Ir film heterostructures and the character of Ag^+^ ion release into solution [26]. In order to explain the differences observed in the present work, we evaluated the dissolution dynamics for Ag/Au heterostructures using the example of analogs Ag/Au/Ti samples obtained via PVD. The studies were carried out in a model medium of sodium-phosphate buffer (pH = 6.8), incubating a series of samples for 2, 4, 6, 8, 24, and 48 h, and controlling the amount of Ag in the solution by a highly sensitive ICP-OES method. In addition, the Au content in the solution was determined to test the possibility of a second antibacterial component, i.e., gold ions [40].

**Figure 8:**
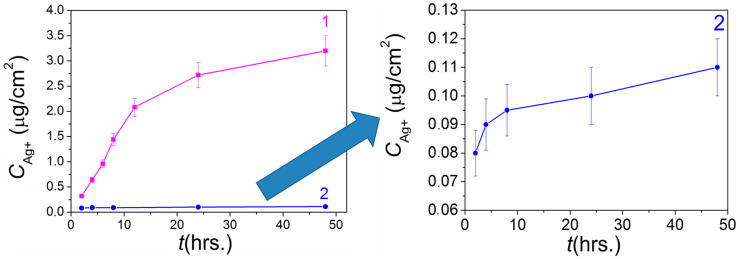
Comparative dynamics of Ag^+^ release from heterostructures Ag/Ir (curve 1) [26] and Ag/Au (curve 2) prepared via PVD onto Ti carriers.

Figure 8 shows the typical dynamics of Ag^+^ release from the surface of Ag/Ir/Ti and Ag/Au/Ti heterostructures with comparable silver content. It is shown that the concentrations of silver dissolved from the surface of Ag/Au/Ti heterostructure vary in a narrow range of 0.08–0.11 µg/cm^2^ in the period of 2–48 h. The dynamics of silver release is poorly expressed (Figure 8). In addition, gold is registered in the solution in a much smaller amount (0.040–0.055 µg/cm^2^) and with a static character of release.

#### 2.3.2. Cell Viability

The in vitro cytotoxicity of Ag/Au/Ti, Ag/Au/CFR-PEEK, Ag/Ir/Ti, and Ag/Ir/CFR-PEEK heterostructures was also investigated to determine the possible influence of the chemical form of Ag and the sublayer (Ir, Au) on cell viability. Each series consists of three experimental samples obtained under the same conditions (See details in Section 4.2).

**Figure 9:**
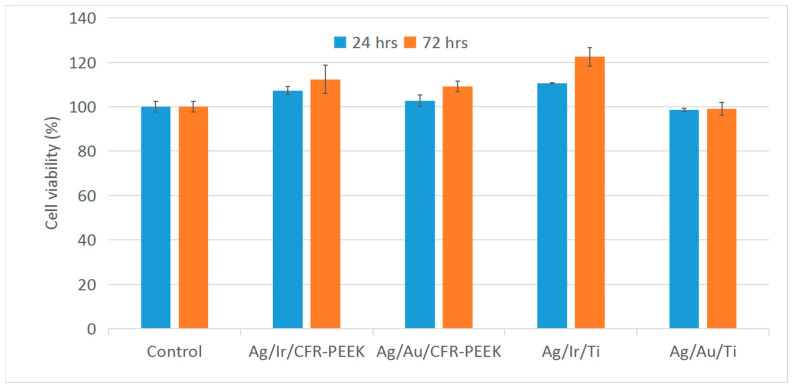
L-929 cell viability in extracts of Ag/Au/Ti, Ag/Au/CFR-PEEK, Ag/Ir/Ti, and Ag/Ir/CFR-PEEK samples.

The study was performed by the indirect method, evaluating the viability of L-929 cells for 24 and 72 h in extracts obtained by incubating samples in culture medium. The results are presented in histograms (Figure 9). All heterostructures showed no toxic effects (cell viability > 95–98%). In addition, extracts from Ag/Ir-type heterostructures promote pronounced cell proliferation. However, samples with a solid Au layer do not show a significant increase in cell proliferation, in contrast to the previously investigated Au nanoparticle heterostructures [24].

#### 2.3.3. In Vivo Biocompatibility

In vivo biocompatibility was evaluated by morphohistological examination of tissues around the samples implanted in the subcutaneous tissue of laboratory rats for 30 and 90 days. For this purpose, samples with Au or Ir layers on Ti and CFR-PEEK with Ag concentration of 13–18 μg/cm^2^ were selected, which showed a pronounced antibacterial effect according to the present and previous studies [26]. To evaluate the effect of the method of forming the active component, silver was prepared on CFR-PEEK-structures via PVD and Ti-structures via MOCVD. The heterostructures were deposited on both sides of the substrates. In the case of Ti, the effect of increasing its initial roughness was considered (Ag/Ir/Ti, Ag/Ir/Ti* samples). The results of the morphohistological analysis are summarized in Table 1.

**Figure 10:**
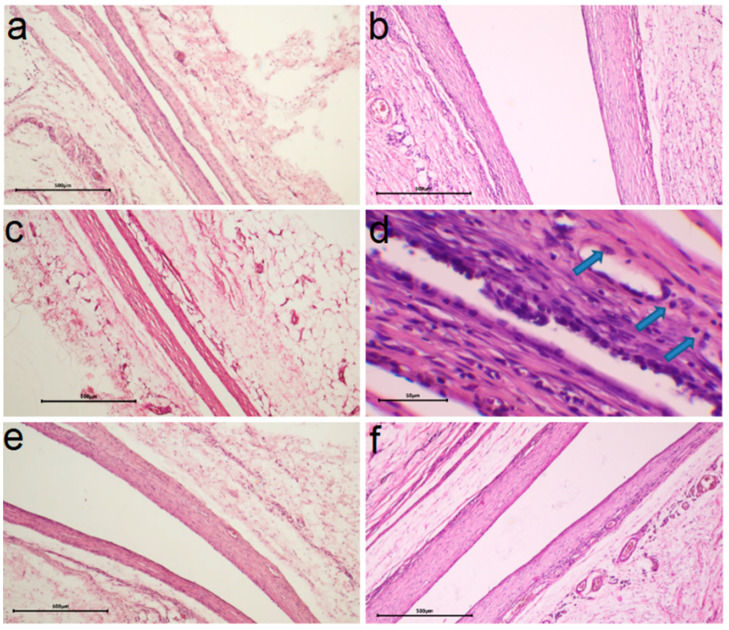
Comparison of histologic capsule structure for Ag/Ir/Ti (**a**), Ag/Ir/Ti* (**c**), Ag/Au/Ti (**e**) specimens after 30 days and Ag/Ir/Ti (**b**), Ag/Ir/Ti* (**d**), Ag/Au/Ti (**f**) specimens after 90 days of implantation (blue arrows indicate macrophages, scale bar is 500 μm for (**a**–**c**,**e**,**f**) and 50 μm for (**d**)).

**Figure 11:**
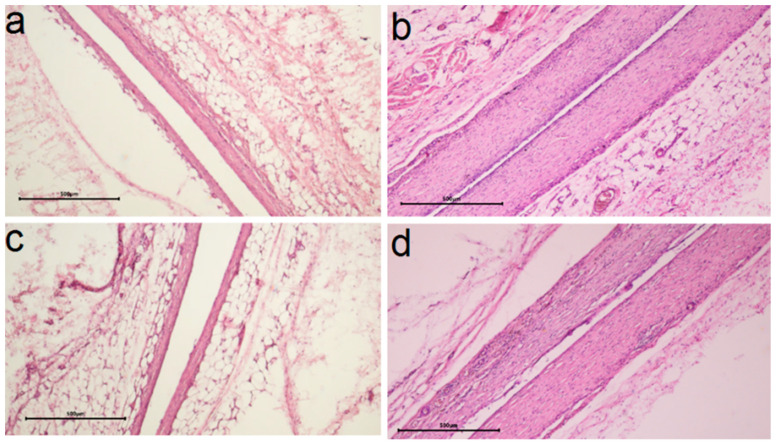
Comparison of histologic capsule structure Ag/Ir/CFR-PEEK (**a**), Ag/Au/CFR-PEEK (**c**) for specimens after 30 days and Ag/Ir/CFR-PEEK (**b**), Ag/Au/PEEK (**d**) after 90 days of implantation (scale bar is 500 μm).

It was found according to the results of morphohistological analysis (Table 1) that after 30 days of implantation, all samples are characterized by the presence of a thin fibrous capsule without volume thickening and signs of inflammation (no lymphocytes, KIT, and mast cells) and with the initial stage of vascular sprouting (Figure 10a,c,e and Figure 11a,c). In addition, for heterostructures on Ti, unlike those on CFR-PEEK, the capsules are characterized by a slight compaction near the contacting surface (hyperchromic stripe). This result is consistent with our previous studies [26] and is a characteristic effect of the carrier material.

After 90 days, most samples show a slight increase in the number of macrophages (an indicator of mild inflammation, possibly argyrosis). In the series of samples obtained via MOCVD (Figure 10 b,d,f), the Ag/Ir/Ti* effect is more pronounced (Figure 10d).

The influence of the sublayer metal (Ir, Au) was more evident in the samples where the active component was obtained via MOCVD (Figure 11a–d). Thus, for the Ag/Au/Ti heterostructure, compared to Ag/Ir/Ti, a denser capsule was formed in 30 days, which slightly decreased in the following 60 days, but no macrophage appearance was observed in the long term (Table 1). In the case of structures obtained via the PVD method, morphohistological characteristics and dynamics of their changes are more comparable (Table 1). An increase in the number of microvessels in the implant contact zone can be observed after 90 days for the Ag/Au/CFR-PEEK specimen (Figure 11c) compared to the Ag/Ir/CFR-PEEK analog (Figure 11c). The saturation of the surrounding tissue with vessels indicates the beginning of the next phase of implant engraftment, and its faster achievement may be due to the rougher surface of these specimens.

To assess the relative nature of silver dissolution from the surface of heterostructures under the dynamic conditions of a living system, quantitative measurement of the Ag content remaining on all samples was performed using ICP-OES. The results are summarized in Table 2. In the case of heterostructures obtained via PVD, the material of the solid layer (Ir or Au) has no influence on the dissolution pattern of silver in the animal body. On the contrary, the Ag/Au/Ti structure obtained by the MOCVD method is fundamentally different from the others: a less intense but more prolonged process is observed. In this case, only 10% of the silver dissolves in 30 days, and then about 20% more in 60 days. In the other samples, most of the Ag (70–90%) is dissolved within 30 days, and the subsequent process is much slower (only ~50% of the remaining Ag is dissolved). Quantitatively, the maximum amount of the active component was dissolved with Ag/Ir/Ti* (for 90 days not less than 23.55 μg), which once again confirms the favorability of the developed surface for the release of silver ions.

**Figure 12:**
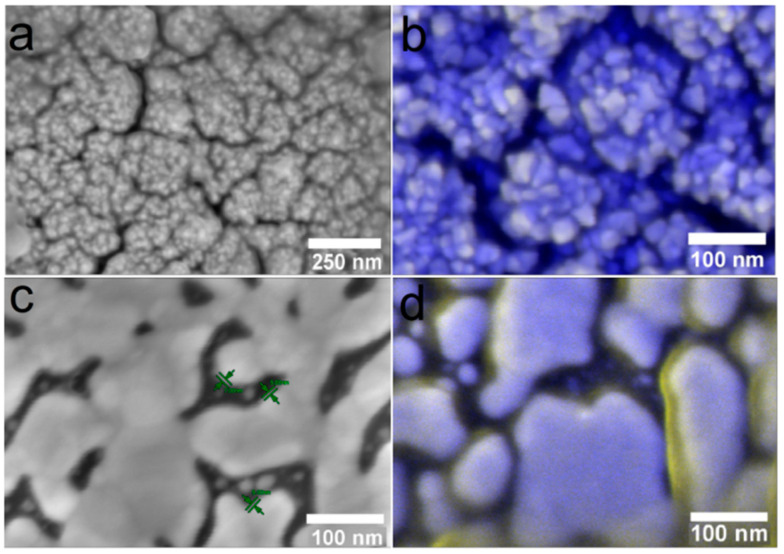
SEM micrographs of heterostructures Ag/Ir/Ti* (**a**,**b**) and Ag/Au/Ti (**c**,**d**) after 30 (**a**,**c**) and 90 (**b**,**d**) days of implantation.

The study of the samples after implantation was supported by SEM data. It was found that regardless of the type of dissolution, it is the Ag film that is preserved on the noble metal surface, while clusters and nanoparticles are absent due to their greater chemical activity (Figure 12). After three months of implantation, this film is most visible on the Ag/Au/Ti sample (Figure 12 d, yellow Ag film, blue Au crystallites), while it is barely visible on the other samples (Figure 12b, yellow Ag film, blue Ir crystallites). Thus, the data from independent SEM and ICP-OES methods harmonize with each other.

## 3. Discussion

### 3.1. Film Heterostructures: Active Component Formation

The literature describes many methods for the production of metal nanoparticles, namely chemical (e.g., chemical reduction, irradiation, electrochemical, microemulsion, etc.), biological (bacteria, fungi, plant extract) and physical (e.g., evaporation, laser ablation, lithography, etc.) [41,42,43,44,45,46]. In this work, among the variety of methods, we have chosen gas-phase methods (PVD and CVD) because they allow to obtain “naked” nanoparticles without the introduction of additional stabilizing agents and foreign chemical compounds, which is important for biological applications. Additionally, they provide precision control of the processes of nucleation and growth of metal layers and the best direct contact of silver with the noble metal sublayers, which ensures the effective action of galvanopairs.

The new data obtained here are consistent with previous results [25] and support the version that the formation of Ag on the surface of noble metals by gas-phase deposition methods is realized according to the well-known mechanism of layer-by-layer growth/mixed layer-island growth [47]. This leads to the fact that the direct formation of thin silver films is observed, on the surface of which isolated nanoparticles and/or clusters are then assembled. In the case of the Au sublayer, these Ag grains are localized mainly at the boundaries of the crystallites, which is probably due to their large size (compared to Ir). During subsequent procedures of silver deposition, particles/clusters combine into larger assemblies, but the thickness of the Ag film increases only in the case of the PVD process. This is probably due to the lower temperature of the substrate [48]. At the same time, the use of sequential PVD and MOCVD procedures looks like a promising approach in terms of controlling the silver content in heterostructures. In the studied samples, the dependences of the amount of deposited silver (according to ISP-OES data) on the number of deposition cycles are close to linear. However, the extension of this interval requires further confirmation and determination of the limitation of these correlations. On the other hand, it is of interest to investigate low concentrations of silver, since it is possible to obtain a discontinuous film/AgNPs in this case [25].

The second important finding is the influence of the noble metal sublayer on the chemical state of the resulting silver. In fact, the formation of silver oxide on the Au surface occurs both during PVD and MOCVD, i.e., at any initial silver oxidation degree (Ag^0^ for PVD (powder) or Ag^+^ for MOCVD ([Ag(cod)(hfac)]_2_ precursor)). This phenomenon is apparently caused by the interaction between the growing Ag film and the Au surface, as a result of which electron transfer from silver to gold takes place [49]. It is worth mentioning that for other noble metals (Ir, Pt) such an effect was not observed, i.e., oxidized silver was not observed in Ag/Ir and Ag/Pt heterostructures [24,25,26]. Thus, for Ag/Au film structures, it is advisable to further investigate in more detail the effect of deposition conditions (primarily temperature) on the ratio of oxidized and metallic forms of silver, as well as to confirm their spatial localization (see Section 3.2).

At the same time, undoubtedly, the formation of the oxide phase in significant concentrations is favored by the chemical activity of silver in the precursor. In particular, the portion of metallic silver in Ag/Au heterostructures is 80% in the case of PVD and 30% in the case of MOCVD. Possibly, incomplete silver reduction occurs under MOCVD experimental conditions due to the existing Ag-O bonds in the [Ag(cod)(hfac)]_2_ precursor. Thus, the MOCVD method seems to be preferable for obtaining mixed Ag-Ag_2_O antibacterial materials. It should be noted that silver oxide, especially in the form of nanoparticles, has not only its own antibacterial effect [50,51] but also a pronounced anti-inflammatory and antifungal effect [52,53,54]. This stimulates active interest in the development of Ag_2_O-containing materials for the surface modification of implants [51,52,55].

### 3.2. Antibacterial Activity

It is important to note that Ag^+^ ions are considered here as the main antibacterial agent released from the surface of all the studied structures. The mechanism of their action on bacteria is still under discussion and includes several aspects: ions can bind to enzymes, causing their inactivation [50] or death due to the accumulation of reactive oxygen species (ROS) [56], as well as have a genotoxic effect on bacterial DNA [57,58]. Despite the variety of assumptions, many researchers have found that the antibacterial activity of materials directly depends on the concentration of Ag^+^ in solution [56].

The observed complete inhibition of bacterial growth after 24 h exposure for all developed heterosystems indicates the effectiveness of the developed antibacterial coatings. At the same time, a significantly higher susceptibility of *P. aeruginosa* compared to *S. aureus* is observed at shorter exposure times (from 2 to 6 h). For example, depending on the sample composition, complete inhibition of *P. aeruginosa* can be achieved within 2 h, whereas for *S. aureus*, it takes 24 h (Table S1, Figure 7). Such resistance to the action of silver is generally characteristic of G+ bacteria and may be related to the peculiarities of the bacterial wall structure. Simultaneous destruction of membrane phospholipids and dissociation of amide bonds and secondary structure of peptidoglycans present in the cell wall structure is more catastrophic for G− bacteria than for G+ bacteria. The simpler single-layer wall structure of G+ bacteria allows them to combat the appearance of “holes” in the membrane more effectively than G− bacteria. Their 2-layer wall is damaged not only in its entire depth but also along its length with the separation of its layers. This seems to explain the better survival of *Staphylococcus aureus* in media rich of silver ions [59].

In general, the differences in the antibacterial activity of the samples during the first hours of incubation seem to be due to differences in the dynamics of the release of Ag^+^ ions into the biological medium. In turn, the oxidative dissolution of Ag is influenced by a combination of factors, including the peculiarities of the chemical state of Ag on the surface (the presence of the oxide phase along with the metallic phase), the form of silver (film/particles/clusters and their sizes) and electrochemical activation (realization of Ag-Ir and Ag-Au galvanopairs).

A comparison of the dynamics of silver dissolution under similar conditions from the surface of similar Ag/Au/Ti (this work) and Ag/Ir/Ti heterostructures [26] shows that silver concentrations released from the Ir surface is more than one order of magnitude higher (0.44–3.3 vs. 0.08–0.11 μm/cm^2^, Figure 8). In addition to the galvanopair effect, this enhanced silver dissolution is due to the higher-developed surface of the Ag/Ir heterostructure.

The significantly slower release of Ag^+^ from the Ag/Au/Ti surface is apparently due to the presence of silver in both the metallic and oxidized states. Both the factor of decreased solubility (Ag_2_O compared with Ag) and the difficulties in realizing the galvanic effect could be taken into account here. The results suggest that the Ag_2_O phase is localized on the surface of the metallic Ag, which prevents the Ag-Au galvanic couple from contacting the solution and thus inhibits the rate of Ag^+^ release. This is reflected in the less pronounced dynamics of the activity of Ag/Au heterostructures against *P. aeruginosa* in short exposure periods compared to Ag/Ir counterparts.

On the other hand, according to the ICP-OES results, the antibacterial action of the second agent, Au^+^, which is also released into the solution, could be suggested for Ag/Au film materials. This could explain the similar dynamics of the effect against *S. aureus* for both types of heterostructures, although the Ag/Au one generates significantly less Ag^+^. A synergistic effect between Ag^+^ and Au^+^ could contribute to the effect of such low concentrations of gold ions [40,60] but this aspect requires further study.

Thus, the activity of Ag/Ir heterostructures is due to high Ag^+^ release rates, which leads to a rapid (2–4 h incubation) inhibition of *P. aeruginosa* growth. In the case of Ag/Au type heterostructures, the growth inhibition of both cultures is slower (from 6 h), with the antibacterial activity possibly due to the contribution of two agents (Ag^+^ and Au^+^ ions). It is also possible that Ag participates in the process of Au dissolution (e.g., due to the formation of a solid solution on the surface of the “solid” layer at the temperatures of Ag deposition) [61,62,63].

### 3.3. In Vivo and In Vitro Biocompatibility

All developed coatings showed the expected lack of cytotoxicity in vitro against a standard fibroblast line. Importantly, improved cell proliferation was observed for most of the extracts. In fact, metal ions are characterized by antiproliferative potential [64], while the stimulation of proliferation is mainly observed for AgNPs [65,66].

The positive effect observed at ion concentrations generated by film heterostructures is valuable for orthopedic implant materials. The ability to manipulate fibroblasts as “stem-like” cells would open an effective gateway for tissue regeneration in the future.

For the first time, the dynamics of silver release into the living organism at the in vivo level was evaluated for such film heterostructures. The key factor is the ratio of metallic and oxidized silver, which is influenced by both the noble metal layer and the method of its deposition (Section 3.1).

In the case of PVD, the Ag^0^ content decreases from 100 to 80% when Ir is replaced by Au, while from 90 to 30% in the case of MOCVD. Accordingly, the picture of silver dissolution for samples with the content of Ag metal phase 100–80% is generally similar and is characterized by the dissolution of the maximum amount of this active component already during 1 month of implantation. This dissolution also leads to a reaction of the organism, i.e., the appearance of macrophages (result after 3 months of implantation). Moreover, for the Ag/Ir/Ti* sample with a developed surface and effective galvanopair, an increased number of macrophages is already observed. Apparently, these are initial or local manifestations of argyrosis [67,68]. On the contrary, the Ag/Au/Ti sample, where the silver oxide phase predominates, showed a less intense and more uniform (almost linear) dissolution of silver. The absence of macrophage appearance at long implantation times found for the Ag/Au/Ti structure confirms the assumption that the prolonged character of the antibacterial component release is preferable. This conclusion is in full agreement with the main result of previous work on heterostructures with other Ag/M/CFR-PEEK (M = Ir, Pt) sublayers [24]. In addition to the previously shown possibility of controlling the release dynamics by the structural organization of the active (silver) phase, the present work demonstrates an alternative way—by controlling the chemical composition of this part of the heterostructure.

In general, summarizing the results of the in vivo studies, it can be said that even a small amount of antibacterial component is sufficient to improve the engraftment of the considered biomaterials due to film heterostructures. However, it is important to emphasize that the implantation experiments were performed in healthy animals and in the absence of hospital-acquired infections. It is likely that the active phase of silver release in the first hours is necessary to prevent infection of a weakened organism or in case of direct bacterial penetration into the wound [69,70]. In this context, the development of film heterostructures with a “mixed” character of silver release seems to be an urgent task.

The presence of Ag/Ag_2_O phases and the release effect of Au^+^ ions make Ag/Au heterostructures promising objects for further investigation. On the other hand, samples with Ir sublayer are still characterized by a promising overall biological effect, controlling the phase composition of the antibacterial component seems to be an effective strategy to control the dynamics of silver release in the case of Ag/Ir heterostructures. For this purpose, the MOCVD method is suitable, where it is possible to vary the concentration of the oxide phase, e.g., by precursor selection or by alternating short-term oxidation/reduction processes.

## 4. Materials and Methods

### 4.1. Materials

The discs made of Ti-alloy and carbon-fiber-reinforced polyetheretherketone assigned as “Ti” (thickness 2 mm, diameter 10 mm, produced by Co., Ltd., Baoji, China) and “CFR-PEEK” (thickness 1–2.5 mm, diameter 20–30 mm, produced by Co., Ltd., Changzhou, Jiangsu, China) were chosen here as carriers for deposition Au, Ir films and corresponding Ag-heterostructures. In addition, the surfaces of Ti-alloy discs were treated by laser resurfacing to increase their roughness giving the substrate further assigned as “Ti*”.

MOCVD precursors were synthesized and purified by us.

The complexes [Ir(cod)(acac)] (>99%, CARLO-ERBA-11008 setup: found: C, 39.0; H, 4.4, calcd. C, 39.1; H, 4.8) and [Ir(CO)_2_(acac)] > 99%, CARLO-ERBA-11008 setup: found: C, 24.5; H, 2.0, calcd.: C, 24.2; H, 2.1) were served as precursors in MOCVD of Ir films [71]. Au powder (>99%, NAZ (Novosibirsk Refinery) Ltd., Novosibirsk, Russia) was used as a source for PVD of Au films.

For PVD and MOCVD of Ag nanomaterials, the following precursors were used: Ag powder (>99%, NAZ (Novosibirsk Refinery) Ltd., Novosibirsk, Russia) and [Ag(cod)(hfac)]_2_ (>99%, CARLO-ERBA-11008 setup: found: C 36.9; H 3.1; F 26.9, calcd.: C 36.9; H 3.0; F 27.1) [72].

For the analysis of heterostructures by inductively coupled plasma atomic emission spectroscopy, the standard solutions of gold (standard solution of gold MSDS, 170216, Merck, Rahway, NJ, USA) and silver (standard solution of silver, MSDS, 119797, Merck, Rahway, NJ, USA) were used.

Pure gases (argon, oxygen, and hydrogen) were provided by Chistye Gases Ltd., Novosibirsk, Russia.

### 4.2. Preparation of Ir, Au Layers and Ag/Ir, Ag/Au Heterostuctures

Immediately before the deposition of heterostructures, the surfaces of Ti, Ti* and CR-PEEK carriers were degreased in isopropanol using an ultrasonic bath for 30 min. The details of MOCVD of Ir films has already been described [25]. In brief, Ir coatings were obtained on Ti and Ti* discs from [Ir(cod)(acac)] at deposition temperature T_d_ = 310 °C, evaporator temperature T_v_ = 110 °C. Ar and O_2_ flow rates were 2 l/h, total pressure *p* = 1 Torr, load = 60 mg, deposition time t = 120 min. Ir coatings were obtained on CFR-PEEK discs from [Ir(CO)_2_(acac)] at T_d_ = 320 °C, T_v_ = 83 °C, Ar, O_2_ = 2 L/h, total pressure *p* = 1 Torr, load = 80 mg, t = 240 min. The Ir layers deposited on Ti, Ti* or CFR-PEEK discs were marked as Ir/Ti, Ir/Ti* or Ir/CFR-PEEK, respectively.

Au coatings were obtained on Ti, CFR-PEEK discs and model Si plates via PVD at the following conditions, UVM.71 installation, U = 500 V, I = 500 mA, *p* = 8 × 10^−7^ Torr, T_v_ = 1670 °C, T_d_ = 200 °C, gold load = 100 mg, giving samples marked as Au/Ti, Au/CFR-PEEK and Au/Si, respectively. The thicknesses of Au layers were estimated by SEM (cross-section of samples on model Si substrates).

Further, the prepared Ir/Ti, Au/Ti, Ir/Ti*, Ir/CFR-PEEK, and Au/CFR-PEEK samples were used as substrates (Table 3, Column 1) to obtain Ag-containing film heterostructures. For this purpose, the surface of these substrates was modified with silver through the PVD or MOCVD processes. Silver deposition was carried out in one, two or three consecutive procedures under the conditions listed in Table 3 (column 6). The dependence of the silver content on the number of deposition experiments and the characteristics of the morphology of typical heterostructures are given in Supporting Information (Figures S1–S3, Supplementary Materials).

The following samples with comparable silver content were selected for biological studies: 1 deposition procedure for MOCVD and 2 deposition procedures for PVD. The detailed characteristics of these samples are presented in Section 2.2.

### 4.3. Methods

The set of standard methods (Table 4) was used for the characterization of the obtained Ir and Au layers and Ag/Ir, Ag/Au heterostructures.

### 4.4. Biological Studies

#### 4.4.1. Antibacterial Activity Test

The biocidal efficacy of the heterostructure samples (Table 3) was evaluated on *S. aureus* and *P. aeruginosa*. The study was performed by the direct contact method as previously described [25], counting surviving bacterial colonies stained with BactoView Live Red kit (Biotium, Fremont, CA, USA), using light microscopy (Axioskop 40FL, Carl Zeiss, Jena, Germany). To evaluate the dynamics of the effect of the heterostructures, bacterial cultures were incubated on the surface of the samples for 2, 4, 6, 24 and 48 h. For control, 24-h incubation of both bacterial colonies in Petri dishes was performed in the absence of heterostructures under similar conditions. Measurements in each experimental group were performed in three replicates.

#### 4.4.2. Cytotoxicity via the XTT Test

Cytotoxicity studies of the samples were performed with extracts of heterostructures. For this purpose, the samples were incubated in DMEM culture medium at 37 °C in a humidified atmosphere with 5% CO_2_ for 72 h at a medium volume to sample surface ratio of 1.25 mL/cm^2^.

L-929 cells were cultured in DMEM medium (Thermo Fisher Scientific, USA) supplemented with 10% fetal calf serum (Thermo Fisher Scientific, USA), 100 U/mL penicillin (Gibco, Waltham, MA, USA), 100 U/mL streptomycin (Gibco, USA), and 2 mmol/L L-glutamine (Invitrogen, Carlsbad, CA, USA) at 37 °C in a humidified atmosphere containing 5% CO_2_. To examine the cytotoxicity of the extracts, cells were seeded in 96-well flat-bottomed culture plates at 1 × 104 cells per 200 μL medium in each well and incubated for 24 h. The medium was then replaced with 200 μL of extract. After incubation in extracts at 37 °C in a humidified atmosphere with 5% CO_2_ under standard conditions for 24 and 72 h, cell viability was measured using the XTT cell proliferation assessment kit (Appli-chem, PanReac Applichem, Barcelona, Spain). The optical density of the well contents was measured at a wavelength of 450 nm and a reference wavelength of 655 nm using an iMark tablet photometer (Bio-Rad Laboratories Inc., Hercules, CA, USA). Cells cultured in DMEM medium were used as controls. All studies were performed in triplicate. Cell viability was calculated as the ratio between the optical density in the experimental groups and the control (A):

Cell viability = (A experimental group/A control) × 100%.

#### 4.4.3. Subcutaneous Implantation in Animals

The in vivo studies of biocompatibility were approved by the local ethics committee of the E. Meshalkin National Medical Research Center of the Ministry of Health of the Russian Federation. The study protocol was conducted in accordance with the recommendations for the proper use and care of laboratory animals (European Communities Council Directive 86/609/EEC and the principles of the Declaration of Helsinki). Wistar rats (weight 130–150 g, n = 8) were used for experiments. The samples were implanted subcutaneously in the back of the experimental animals according to conventional methods under anesthesia in the operating room, in compliance with the rules of asepsis and antisepsis. The animals were observed in the vivarium for 30 or 90 days before being euthanized for histological studies.

#### 4.4.4. Histological Test

To study the morphologic structure of the tissue surrounding the implants, the implants were removed together with the surrounding connective tissue capsule after 30 or 90 days. The obtained specimens were fixed for 48 h in a 20-fold excess (by volume) of 10% buffered formalin. The implants were then carefully removed, and the fibrous capsule was subjected to histologic processing and embedded in paraffin blocks. Five-micrometer sections were prepared from the paraffin blocks and stained with hematoxylin-eosin according to the generally accepted technique. Histological analysis was performed using an Axioskop 40FL microscope (Carl Zeiss, Germany) equipped with an ADF Pro 08 color camera andADF ImageCapture version 1.0 software (all ADF Optics, Shenzhen, China). A scoring system (from 0 to 3) was used to describe the morphohistological findings, where 0 was the best score and 3 was the worst score. The following parameters were scored: packing density, microvessels, macrophages, lymphocytes, foreign body cells (FBC), and mast cells.

Quantitative data were processed using Statistica 13.0 (TIBCO Software Inc., Palo Alto, CA, USA). The normality of the datasets in each experiment was tested using the Shapiro–Wilk test. Student’s *t*-test was used to detect differences between groups. Statistical significance was set at *p* < 0.05.

## 5. Conclusions

Ag/Au and Ag/Ir film heterostructures on the surface of modern Ti and CFR-PEEK implant materials were prepared using gas-phase methods, viz. PVD and CVD. It was found that upon deposition of silver as a bactericidal component, its formation on the surface of noble metals occurs by the mechanism of layer-by-layer/mixed layer-island growth. In the case of the Au sublayer of the heterostructure, regardless of the deposition method, not only metallic but also oxidized silver is formed, which is probably due to the transfer of electrons from silver to gold.

For all heterostructures, the absence of in vitro cytotoxic effects on human fibroblasts was shown (24 and 72 h). The biocidal efficacy of the prepared heterostructures was evaluated on *S. aureus* and *P. aeruginosa*. Complete inhibition of the growth of both bacterial colonies after 24 h exposure was established. The differences in the composition of the samples are reflected in their antibacterial activity in a short incubation period (1–6 h), which is apparently due to the dynamics of Ag^+^ release into the biological medium. Ag^+^ release is more pronounced for Ag/Ir heterostructures than for Ag/Au counterparts, which is due to the presence of oxidized silver in the latter case. However, for Ag/Au heterostructures, a slow dissolution of gold, which can also have a specific antibacterial effect, is observed. It was found according to the results of a morphohistological study that a thin fibrous capsule without volume thickening and signs of inflammation is formed after 30 days of implantation of the heterostructure samples. After 90 days, a slight increase in the number of macrophages (an indicator of mild inflammation or manifestation of argyrosis), which is the maximum for samples of Ag/Ir with the most active Ag^+^ release, occurred. At the same time, such an effect was not observed in the case of Ag/Au heterostructures due to the delayed dissolution of silver. In general, it has been shown that a small amount of the antibacterial component was sufficient to increase the survival rate of the biomaterials under consideration due to film heterostructures based on noble metals. The obtained results open up new ways to optimize the composition of such materials to improve the biological properties of modern implant materials.

## Figures and Tables

**Table 1:** Summary of findings from histological examination of materials.

Sample	Implantation Time (Days)	Packing Density	Microvessels	Macrophages	Lymphocytes	FBCs	Mast Cells
Ag/Ir/Ti	30	1–2	1	1	0	0	0
	90	1	1	1	0	0	0
Ag/Ir/Ti*	30	0–1	1	0	0	0	0
	90	2–3	1	2–3	0	0	0
Ag/Au/Ti	30	2	1	0	0	0	0
	90	1–2	0	0	0	0	0
Ag/Au/CFR-PEEK	30	0–1	1	0	0	0	0
	90	1–2	2	1	0	0	0
Ag/Ir/CFR-PEEK	30	1–2	1	0	0	0	0
	90	1	1	1	0	0	0

**Table 2:** Silver content (μg/cm^2^) in heterostructure samples before and after 30 days and 90 days of implantation.

Sample	Initial Ag Content (μg/cm^2^)	Implantation Time (30 Days)	Implantation Time (90 Days)
Ag/Au/CFR-PEEK	13.2 ± 1.3	1.8 ± 0.1	0.89 ± 0.08
Ag/Ir/CFR-PEEK	13.5 ± 1.3	1.4 ± 0.1	0.89 ± 0.08
Ag/Ir/Ti	15.7 ± 1.5	5.2 ± 0.2	2.6 ± 0.1
Ag/Ir/Ti*	16.2 ± 1.5	3.2 ± 0.2	1.4 ± 0.1
Ag/Au/Ti	18 ± 1.5	16 ± 1	13.2 ± 0.3

**Table 3:** Deposition conditions of Ag/Ir and Ag/Au heterostructures.

Substrate	Deposition Conditions					Sample Series
	Precursor	Load (mg)	Temperature (°C)		Details	
			T_v_	T_d_		
CFR-PEEK	Ag	5	1300	150	PVD UVM.71 installation, U = 400 V, I = 400 mA 8 × 10^−7^ Torr	Ag/CFR-PEEK
Ir/CFR-PEEK						Ag/Ir/CFR-PEEK
Au/CFR-PEEK						Ag/Au/CFR-PEEK
	[Ag(cod)(hfac)]_2_	40	120	300	MOCVD hand-made installation, v(Ar) = 9 L/h, v(H_2_) = 12 L/h, 5.7 Torr	
Ir/Ti						Ag/Ir/Ti
Ir/Ti*						Ag/Ir/Ti*
Au/Ti						Ag/Au/Ti

**Table 4:** Methods used for the sample characterization and their experimental details.

Method	Abbreviation	Equipment and Experimental Details	Interpretation
X-ray diffraction	XRD	Shimadzu XRD-7000 diffractometer, Shimadzu, Kyoto, Japan, CuKα radiation (2θ = 10–80°).	XRD patterns of Au/Ti and Au/CFR-PEEK samples were indexed according to the RDF-2 database (ICDD, Newtown Square, PA, USA) (version 2022).
Inductively coupled plasma atomic emission spectroscopy	ICP-OES	High-resolution spectrometer iCAP 6500 (Thermo Fisher Scientific, Waltham, MA USA). The registration of samples was performed at the axial observation of plasma: cooling argon flow was 12 L/min, secondary was 0.5 L/min, registration time was 5 s, power supplied to an ICP inductor was 1150 W.	The nitric acid solutions of as-deposited samples (Table 3, Column 7) and samples after 30 and 90 days of implantation were prepared using the standard procedure described in Ref. [25]. The Ag and Au contents were calculated using the most intense analytical lines: 208.209, 242.795, 267.595 nm for Au, and 328.068, 338.289 nm for Ag.
X-ray photoelectron spectroscopy	XPS	PHOIBOS-150 analyzer with 1D-DLD detector, FOCUS-500 monochromator (SPECS, Berlin, Germany), Al Kα radiation, hv = 1486.71 eV, 14 kV, 200 W). Calibration of binding energies by the Fermi level of the valence band (0.0 eV).	The spectra of Ag/Au/CFR-PEEK, Ag/Ir/Ti, and Ag/Au/Ti samples were processed in the CASA program version 2.2 software (Tokyo, Japan) using the Voigt function. The background was taken into account using the Shirley method. For Ir, Au 4f peaks fitting, the parameters proposed by Pfeifer et al. were used [73].
Atomic force microscopy	AFM	Microscope with a semi-contact mode with an Ntegra Prima II (NTMDT, Moscow, Russia): length was 123 µm, width was 34 µm, force constant was 17 N/m, and resonance frequency was 230 kHz.	The roughness parameters of bare Ti, Ti* substrates and samples (Table 3, Column 7) were calculated using the Nova SPM version 4.0 software 197374, St. Petersburg, Savushkina street, building 83 k3, office 236, Russia
Scanning Electron Microscopy	SEM	HITACHI UHR FE-SEM SU8200, Hitachi, Ltd., Hitachi, Japan	All samples (Table 3, Column 7) were scanned at the same conditions (3 keV, LA detector). SEM images of the samples (Tiff files) were imported into the program image analysis version 1.54 software (ImageJ) National Institutes of Health, 9000 Rockville Pike, Bethesda, Rockville, MA, USA (version 2.0.0) to study Ag particles size distribution in case of Ag/CFR-PEEK.
Energy-dispersive X-ray spectroscopy	EDX	EDX-analizator EX-2300BU connected with SEM, JEOL-ISM 6700 F microscope, Tokyo, Japan	EDX spectra of Ag/Ir/CFR-PEEK and Ag/Au/CFR-PEEK were recorded using Quantax 70 Bruker version 1.3 software, Schwazchid st. 12, Berlin, Germany

## Data Availability

The data presented in this study are available in this article or on request from the corresponding author.

## Supplementary Materials

The supporting information can be downloaded at: https://www.mdpi.com/article/10.3390/ijms25021100/s1.
