# Supplementary material for: Vapor-Phase-Deposited Ag/Ir and Ag/Au Film Heterostructures for Implant Materials: Cytotoxic, Antibacterial and Histological Studies
Source: Int J Mol Sci. 2024 Jan 16;25(2):1100. doi: 10.3390/ijms25021100 (PMC10816904; doi:10.3390/ijms25021100)
Supplement: Supplementary file 1 [file ijms-25-01100-s001.zip › ijms-2822845-supplementary.pdf]

SUPPORTING INFORMATION

Vapor-Phase-Deposited Ag/Ir and Ag/Au Film Heterostructures for Implant Materials: Cytotoxic, Antibacterial and Histological Studies

David S. Sergeevichev [1,2], Svetlana I. Dorovskikh [1], Evgeniia S. Vikulova [1], Elena V. Chepeleva [2], Maria B. Vasiliyeva [2,3], Tatiana P. Koretskaya [1], Anastasiya D. Fedorenko [1], Dmitriy A. Nasimov [4], Tatiana Y. Guselnikova [1], Pavel S. Popovetsky [1], Natalya B. Morozova [1] and Tamara V. Basova [1,*]

[1] Nikolaev Institute of Inorganic Chemistry SB RAS, 3 Lavrentiev Ave., Novosibirsk 630090, Russia
[2] NMRC Named after Academician E.N. Meshalkin of the Ministry of Health of the Russian Federation, 15, Rechkunovskaya St., Novosibirsk 630055, Russia
[3] V. Zelman's Institute of Medicine and Psychology, Novosibirsk State University, 2, Pirogov St., Novosibirsk 630090, Russia
[4] Rzhanov Institute of Semiconductor Physics SB RAS, 13 Lavrentiev Ave., Novosibirsk 630090, Russia
* Correspondence: basova@niic.nsc.ru; Tel.: +7-383-330-9556

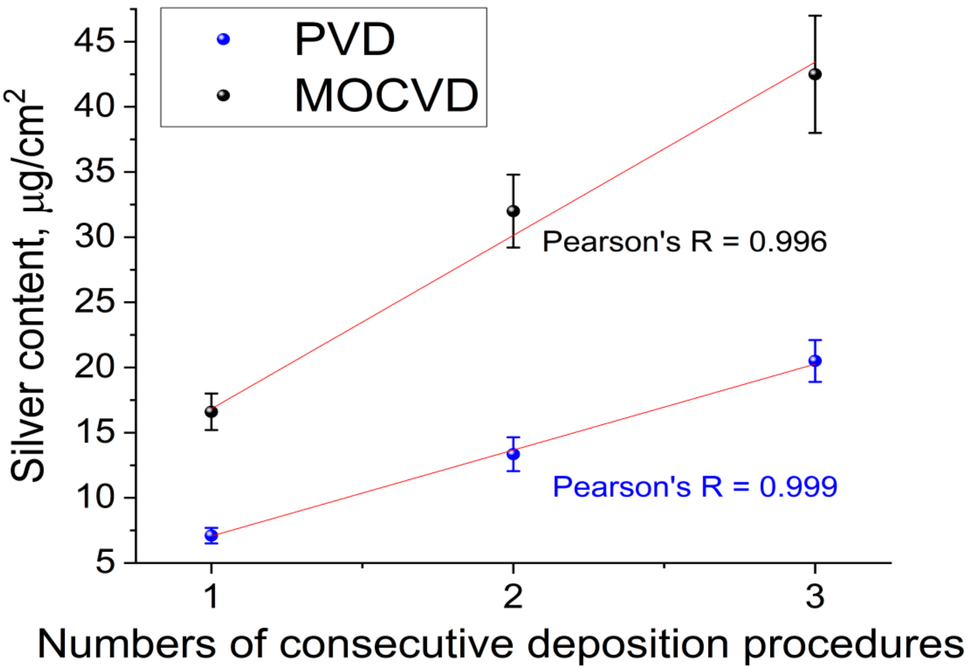

Figure S1. Linear dependencies of Ag content in the Ag/Au heterostructures from the numbers of consecutive (PVD or MOCVD) depositions

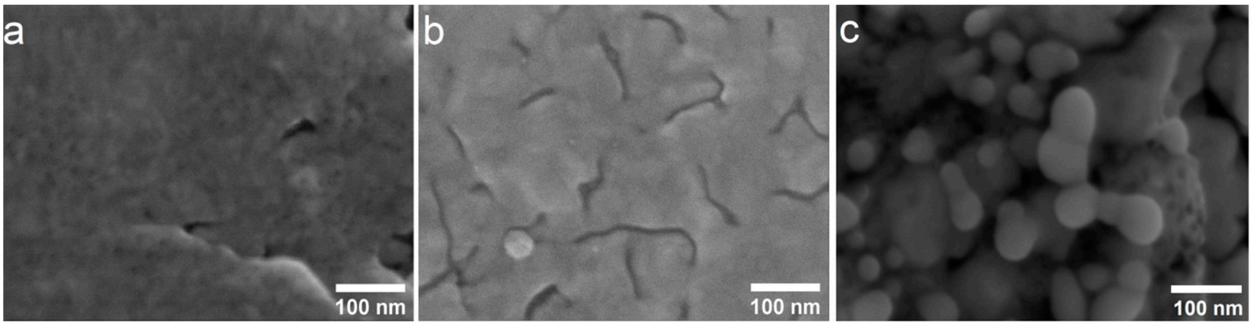

Figure S2. SEM images of Ag/Au heterostuctures obtained on Ti discs by PVD: one consecutive deposition (a), two consecutive depositions (b) and three consecutive depositions (c)

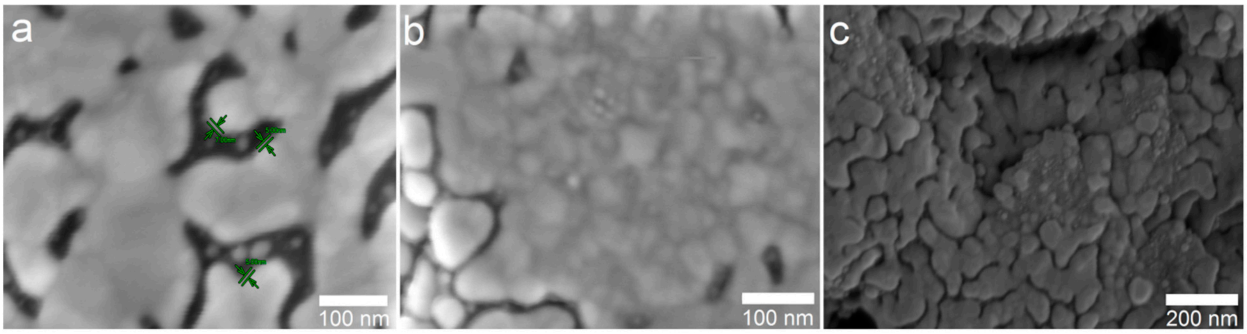

Figure S3. SEM images of Ag/Au heterostuctures obtained on Ti discs by MOCVD: one consecutive deposition (a), two consecutive depositions (b) and three consecutive depositions (c)

Table S1. Quantitative growth indicators of *P. aeruginosa* and *S. aureus* colonies on the studied heterostructure samples.

**Sample group**	***P. aeruginosa***						***S. aureus***					
		2hrs	4hrs	6hrs	24hrs	48hrs		2hrs	4hrs	6hrs	24hrs	48hrs
**Ag/Au/Ti**	Sample 1	941	588	112	0	0	Sample 1	1020	788	193	0	0
	Sample 2	884	425	234	0	0	Sample 2	955	813	278	0	0
	Sample 3	874	641	149	0	0	Sample 3	939	874	291	0	0
	Mean	899.7	551.3	165.0	0.0	0.0	Mean	971.3	825.0	254.0	0.0	0.0
	SD	36.1	112.6	62.6	0.0	0.0	SD	42.9	44.2	53.2	0.0	0.0
**Ag/Ir/CFR-PEEK**	Sample 1	893	118	0	0	0	Sample 1	948	887	482	0	0
	Sample 2	977	203	0	0	0	Sample 2	962	738	368	0	0
	Sample 3	1015	162	0	0	0	Sample 3	1033	791	416	0	0
	Mean	961.7	161.0	0.0	0.0	0.0	Mean	981.0	805.3	422.0	0.0	0.0
	SD	62.4	42.5	0.0	0.0	0.0	SD	45.6	75.5	57.2	0.0	0.0
**Ag/Ir/Ti**	Sample 1	892	0	0	0	0	Sample 1	1009	991	257	0	0
	Sample 2	723	0	0	0	0	Sample 2	1012	1003	322	0	0
	Sample 3	812	0	0	0	0	Sample 3	985	939	239	0	0
	Mean	809.0	0.0	0.0	0.0	0.0	Mean	1002.0	977.7	272.7	0.0	0.0
	SD	84.5	0.0	0.0	0.0	0.0	SD	14.8	34.0	43.7	0.0	0.0
**Ag/Ir/Ti***	Sample 1	0	0	0	0	0	Sample 1	1043	955	384	0	0
	Sample 2	0	0	0	0	0	Sample 2	1001	912	567	0	0
	Sample 3	0	0	0	0	0	Sample 3	990	967	552	0	0
	Mean	0.0	0.0	0.0	0.0	0.0	Mean	1011.3	944.7	501.0	0.0	0.0
	SD	0.0	0.0	0.0	0.0	0.0	SD	28.0	28.9	101.6	0.0	0.0

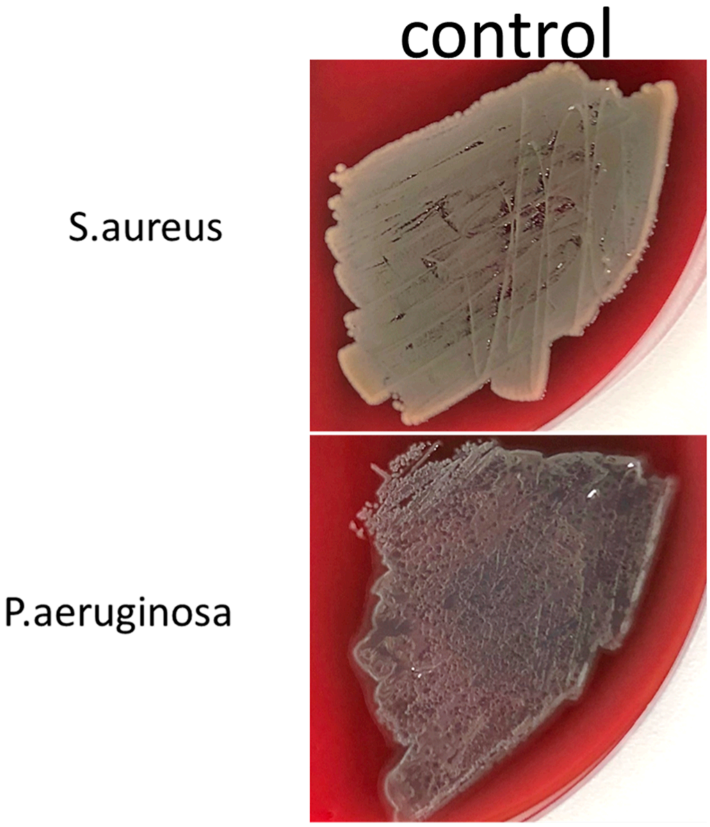

Figure S4. *S. aureus* and *P. aeruginosa* activity profile after 24 hours incubation without investigated heterostructures.
